# Stress-driven remodeling of antigen presentation and chemokine signaling in pancreatic β-cells: implications for type 1 diabetes

**DOI:** 10.3389/fimmu.2026.1772399

**Published:** 2026-04-23

**Authors:** Rahul Mittal, Farhad Alipour, Jhanvi Doshi, Mannat Mittal, Khemraj Hirani

**Affiliations:** 1 Diabetes Research Institute, University of Miami Miller School of Medicine, Miami, FL, United States; 2 Division of Endocrinology, Diabetes, and Metabolism, Department of Medicine, University of Miami Miller School of Medicine, Miami, FL, United States

**Keywords:** antigen presentation, cell stress, chemokine signaling, pancreatic beta cells, type 1 diabetes

## Abstract

Type 1 diabetes (T1D) has historically been framed as a disease initiated and maintained by dysregulated immunity that targets insulin producing β-cells. However, recent findings from human tissue analysis, single cell transcriptomics, and longitudinal cohort studies reveal that intrinsic β-cell stress responses contribute substantially to early disease development. These responses include endoplasmic reticulum stress, remodeling of the unfolded protein response, oxidative and metabolic strain, impaired proinsulin folding and processing, altered granule biogenesis, increased production of cytokines and chemokines, and significant enhancement of antigen presentation pathways. Together, these stress responses create a cellular environment that increases immunogenicity and influences the recruitment and activation of immune cells. This perspective provides a comprehensive integration of mechanistic and clinical evidence showing that β-cell intrinsic biology interacts closely with immune dysregulation to shape disease trajectory. Mechanistic insights from human islets are integrated with translational data from longitudinal clinical studies, revealing a coherent model in which β-cell stress appears early, informing biomarker patterns, influences disease heterogeneity, and provides promising therapeutic targets. This overview offers a unified, balanced conceptual framework to guide future research, early detection strategies, and treatment development.

## 1 Introduction

Type 1 diabetes (T1D) has traditionally been described through the lens of autoimmune pathology, with the immune system viewed as the primary initiator of pancreatic β-cell destruction (1–7). For decades, this interpretation dominated the field and shaped both experimental research and therapeutic development (8, 9). According to the classical model, T lymphocytes with aberrant specificity gain access to the islet environment (10), recognize their targets, and initiate a progressive cascade of inflammatory injury (3, 11, 12). Within this framework, β-cells were portrayed as largely passive participants whose survival depended entirely on the degree of immune dysregulation (13, 14). Although this perspective captured essential immune mechanisms, accumulating evidence from human pancreatic biology now reveals that the earliest molecular alterations occur within β-cell itself, well before clinically evident glucose disturbances and prior to extensive cellular infiltration (15–22). These findings challenge a unidirectional view of disease initiation and place the β-cell, rather than the immune cell, at the center of the earliest pathogenic landscape.

Recent technological advances have been central to this shift in understanding. High resolution imaging of organ donor pancreas, refined single cell transcriptomic analysis, proteomic mapping of human β-cells (9), and reproducible biomarker signatures obtained from longitudinal cohorts consistently demonstrate that the β-cell begins to exhibit distress long before metabolic dysfunction becomes measurable (23–27). These early changes are not subtle variations in physiological responsiveness but substantial alterations in protein folding capacity, mitochondrial metabolism (28), granule biogenesis (29), vesicle trafficking (29), redox homeostasis, and antigen presentation (30, 31). Such alterations indicate that the β-cell undergoes a profound reorganization of its internal environment during the earliest stages of disease, in a manner that creates vulnerability to immune recognition while also generating signals that can influence immune behavior (31). The coherence of these findings across independent platforms and distinct patient populations suggests that β-cell intrinsic stress responses are not incidental to the pathogenesis of T1D but fundamental contributors that shape both cellular fate and disease tempo.

Longitudinal studies from birth cohorts and relatives of affected individuals demonstrate that biomarkers of β-cell stress (32, 33), such as elevations in the proinsulin to C peptide ratio and subtle impairments in glucose stimulated insulin secretion, emerge years before clinical onset (34, 35). These biomarkers mirror the transcriptional and organellar disturbances identified in human pancreatic tissue (36). As a result, the earliest phase of T1D cannot be fully understood as an immunological phase alone, but rather as a period of dynamic interaction between β-cell intrinsic pathways and the evolving immune environment (37).

The relevance of β-cell intrinsic biology extends far beyond molecular endocrinology (38, 39). The concept that stressed endocrine tissues can shape immune activity resonates with observations from other organ specific autoimmune diseases (40, 41), including autoimmune thyroiditis and adrenalitis (42), in which intrinsic alterations in hormone producing cells appear to precede the arrival of pathogenic lymphocytes (43–45). These parallels indicate that endocrine susceptibility to immune recognition may arise, in part, from the unique metabolic and proteostatic demands placed upon hormone secreting cells (38, 46). The β-cell, which sustains one of the highest biosynthetic rates of any differentiated human cell type, is particularly prone to disruptions in protein folding and mitochondrial metabolism (29, 47, 48). These physiological features create a distinct vulnerability profile that can amplify the impact of environmental, metabolic, or inflammatory stimuli (39, 46). The β-cell therefore serves as an ideal endocrine model for understanding how intrinsic cellular strain may interact with immune pathways to initiate organ specific autoimmunity (47).

Framing T1D through this integrated perspective requires synthesis of a diverse range of scientific domains, including endoplasmic reticulum biology (49), mitochondrial metabolism (38), vesicle trafficking (50), immunology (51, 52), clinical epidemiology (53), and the study of preclinical natural history (22). Yet, when these domains are considered together, a coherent narrative emerges (54, 55). β-cells under stress undergo alterations that compromise secretory fidelity (56), increase production of inflammatory mediators (57, 58), broaden antigenic visibility (51), and amplify their own susceptibility to immune mediated injury (53, 59). These cellular alterations align with biomarker trajectories observed in individuals who progress toward clinical disease (60–62), and they provide mechanistic explanations for the timing and heterogeneity of disease onset (63). Importantly, these findings do not diminish the centrality of immune dysregulation but expand the framework in which immune mechanisms operate. Autoreactive T lymphocytes remain essential for β-cell destruction (9, 64), yet their activation and recruitment may be shaped by stress induced features of the β-cell itself (65–67). In this model, the earliest determinants of clinical progression arise from the dynamic intersection of endocrine cell vulnerability and immune activation (68), rather than from immune dysfunction alone.

**Figure 1:**
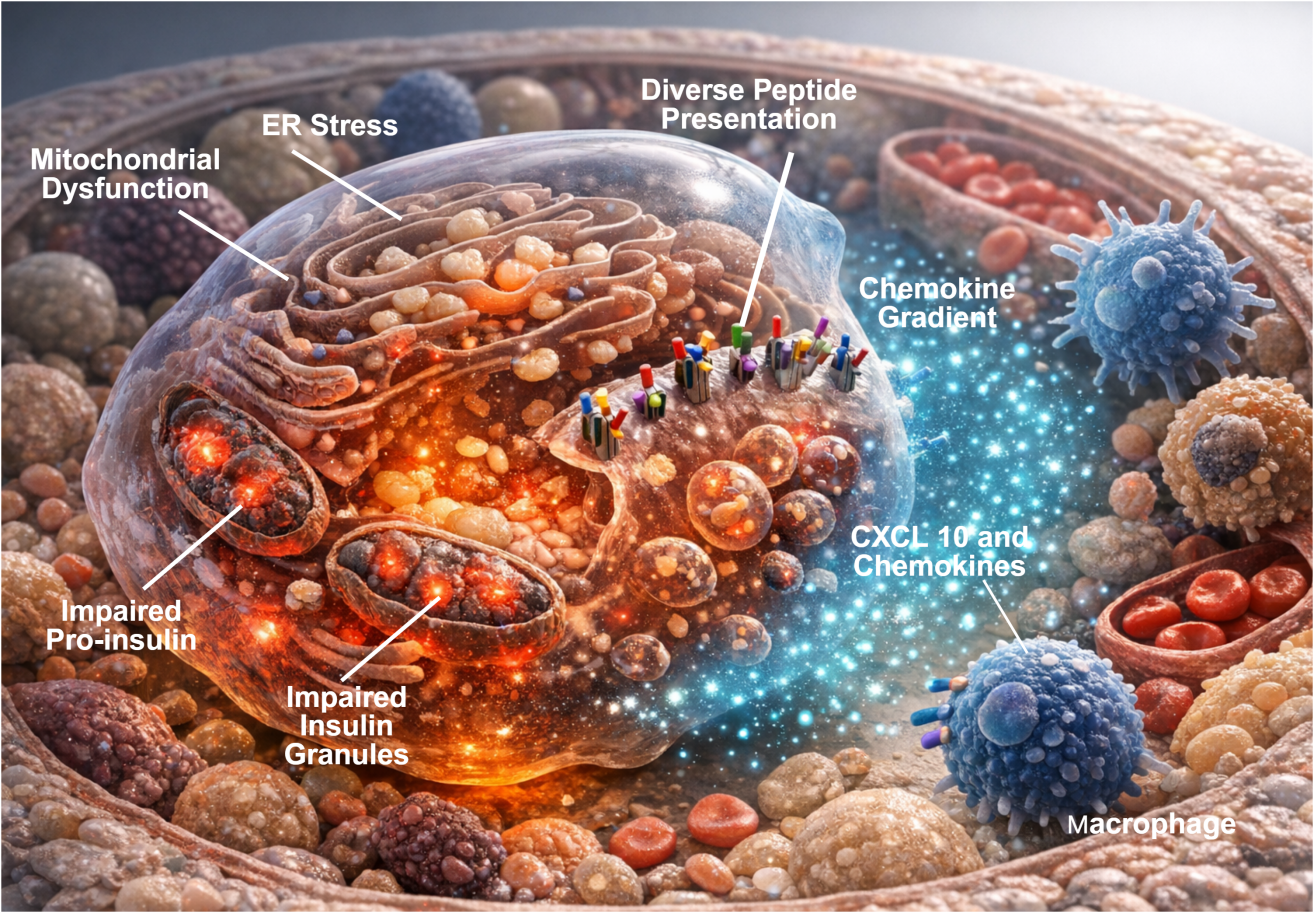
Stress-driven immunogenic reprogramming of the human pancreatic β-cell. Intrinsic stress responses within pancreatic β-cells reshape antigen presentation and chemokine signaling prior to overt immune infiltration. Endoplasmic reticulum stress, mitochondrial dysfunction, and impaired insulin granule biogenesis converge to disrupt proteostasis and metabolic homeostasis, leading to accumulation of misfolded proinsulin and diversification of the β-cell immunopeptidome. These stress-associated peptides are increasingly presented on major histocompatibility complex class I molecules at the β-cell surface, enhancing immunogenic visibility. In parallel, stressed β-cells secrete chemokines, including CXCL10, generating spatial gradients that promote recruitment and positioning of antigen-experienced immune cells within the islet microenvironment. Together, these coordinated β-cell–intrinsic processes establish a permissive immunological niche that precedes and potentiates adaptive immune engagement, positioning the β-cell as an active determinant of early autoimmune activation in type 1 diabetes. Created in BioRender. Mittal, R. (2026) https://BioRender.com/pipjskd.

This perspective seeks to articulate this modern, integrative view of T1D by weaving together mechanistic discoveries from human β-cell research with clinical observations from large longitudinal cohorts. The synthesis presented here builds a unified conceptual structure that encompasses the earliest cellular events within the β-cell (25, 69), the initial immune responses they evoke (70), and the progressive interactions that culminate in clinical T1D (9, 66). By bringing these domains into alignment, this perspective provides a framework that can inform early detection strategies, guide the interpretation of mechanistic biomarkers, and support the design of therapeutic interventions that target the earliest drivers of disease (16). Such an approach recognizes the interdependence of endocrine biology and immune function and places β-cell stress responses at the forefront of contemporary T1D research. These emerging concepts are summarized schematically in **Figure 1**, which shows how β-cell intrinsic stress alters intracellular organization and promotes immune engagement.

## 2 β-cell stress across disease stages

T1D is currently conceptualized as a staged disorder progressing from asymptomatic autoimmunity to dysglycemia and ultimately to symptomatic hyperglycemia (33, 71–74). Stage 1 is defined by the presence of two or more islet autoantibodies in individuals with normoglycemia (75, 76). Stage 2 includes persistent autoimmunity with measurable dysglycemia in the absence of clinical symptoms (77). Stage 3 represents clinical diabetes characterized by symptomatic hyperglycemia and substantial impairment of functional β-cell mass (41). Framing β-cell intrinsic biology within this staging paradigm allows a more structured consideration of when specific stress associated mechanisms are detectable and how they may change over time (**Supplementary Table 1**).

In Stage 1, individuals demonstrate established islet autoimmunity while maintaining normoglycemia (78). Although metabolic parameters remain within the normal range, several lines of evidence suggest that molecular features of β-cell stress may already be present. Transcriptomic analyses of islets from autoantibody positive donors have revealed activation of unfolded protein response pathways, induction of interferon responsive genes, and early modulation of antigen processing components (28, 79–81). In parallel, longitudinal cohort studies have shown that elevations in the proinsulin to C peptide ratio can be detected in individuals who subsequently progress, indicating subtle impairment in proinsulin maturation and proteostasis (32–34, 82, 83). These observations support the possibility that Stage 1 represents a phase of compensated β-cell stress in which intrinsic perturbations precede overt metabolic dysfunction. At the same time, the number of available human samples at this stage remains limited. Additional longitudinal and mechanistic studies are warranted to clarify the reproducibility, magnitude, and functional consequences of these early β-cell stress signatures.

Stage 2 is characterized by the development of dysglycemia in the setting of persistent autoimmunity (53). At this stage, functional abnormalities become measurable, including impaired glucose stimulated insulin secretion and progressive increases in the proinsulin to C peptide ratio (33, 34). These changes are consistent with sustained disruption of proinsulin processing, granule biogenesis, and organellar homeostasis (32, 84–86). Experimental and human tissue data further indicate enhanced expression of MHC-I molecules and components of the antigen processing machinery under inflammatory conditions, suggesting increased β-cell immune visibility (87, 88). Collectively, these findings are compatible with an interval in which intrinsic stress responses become more pronounced and may interact more directly with adaptive immune mechanisms. However, further studies integrating immunopeptidomics, spatial profiling, and metabolic phenotyping across well-defined Stage 2 cohorts will be required to more precisely define causal relationships and temporal sequence.

Stage 3 represents overt diabetes with symptomatic hyperglycemia and significant loss of functional β-cell mass (89–91). At diagnosis, pancreatic tissue demonstrates robust inflammatory signaling, marked upregulation of interferon responsive pathways, and extensive remodeling of antigen presentation networks (80, 92, 93). Many of these alterations likely reflect the cumulative effects of ongoing immune mediated injury superimposed on earlier intrinsic stress pathways (94, 95). In this context, the molecular features observed at Stage 3 can be interpreted as advanced manifestations of processes that may have been initiated during earlier stages (11, 96). Nevertheless, distinguishing primary stress drivers from secondary consequences of inflammation remains an important objective, and additional functional studies in human tissue will be necessary to resolve these relationships with greater precision.

## 3 Early β-cell stress as a driver of disease initiation

### 3.1 Endoplasmic reticulum stress as an early feature of β-cell instability

The β-cell supports an exceptionally high demand for insulin biosynthesis (66, 97). The endoplasmic reticulum is the central organelle responsible for folding, oxidizing, and processing large quantities of proinsulin (98–100). Under physiological conditions, the endoplasmic reticulum maintains delicate homeostasis through a complex proteostasis network that includes molecular chaperones (101), oxidative folding enzymes, and finely regulated calcium dependent protein quality control systems (102–106).

A substantial body of evidence from human islets shows that early activation of the unfolded protein response occurs in β-cells from individuals with autoantibody positivity or recent onset T1D (16, 28). When activation remains prolonged, these pathways shift from promoting adaptation to inducing maladaptive responses (16). These maladaptive responses include increased protein misfolding (107), diminished folding capacity (55), impaired secretory granule formation (108), and increased susceptibility to apoptosis (109).

Endoplasmic reticulum stress within β-cells also amplifies sensitivity to inflammatory cytokines (16, 110). Cytokines such as interferon gamma and interleukin one beta synergize with preexisting endoplasmic reticulum stress to intensify calcium dysregulation (111, 112), oxidant formation, mitochondrial dysfunction (113), and engagement of proapoptotic pathways (3, 114). Human tissue studies show that markers of endoplasmic reticulum stress appear before extensive immune infiltration (7, 115, 116), indicating that these alterations precede and potentially contribute to subsequent immune activation (117). This observation is consistent across multiple independent cohorts and reinforces the importance of β-cell intrinsic pathways during early disease development (**Supplementary Table 1**).

### 3.2 Mitochondrial metabolism and oxidative stress as amplifiers of early dysfunction

β-cells rely heavily on mitochondrial metabolism to generate ATP, which serves as a critical signal coupling glucose metabolism to insulin secretion (118–122). Mitochondria are central regulators of cellular energy status and redox homeostasis (123–125). In the context of elevated secretory burden or inflammatory exposure, mitochondrial metabolic pathways can become strained (126), resulting in increased production of reactive oxygen species (127, 128). β-cells express lower levels of antioxidant defense enzymes relative to many other cell types, rendering them highly susceptible to oxidative injury (129–131).

Studies of human β-cells exposed to cytokines reveal that oxidative stress can disrupt mitochondrial membrane potential, decrease respiratory chain efficiency (132), and impair ATP production (133, 134). These disturbances have profound consequences for insulin secretion (126, 135). Oxidant signals also influence the processing of proinsulin, exacerbate misfolding, and intensify endoplasmic reticulum stress through calcium dependent signaling pathways (73, 136–138). Observations from human pancreatic tissue demonstrate increased oxidative damage in β-cells from individuals with early T1D (139–142). These findings confirm that the oxidative environment within β-cells changes well before overt metabolic dysfunction.

Cross talk between mitochondria and the endoplasmic reticulum further amplifies these effects (138, 143, 144). Perturbations in one compartment can destabilize the other. For example, mitochondrial dysfunction increases calcium release from the endoplasmic reticulum, while unfolded protein response activation alters mitochondrial metabolism (137, 145, 146). These reciprocal influences contribute to a state of sustained cellular stress and vulnerability.

### 3.3 Vesicle biogenesis, granule trafficking, and loss of secretory fidelity

The formation of mature insulin granules depends on coordinated steps involving proinsulin folding, trafficking through the Golgi apparatus, condensation within immature granules (55, 147, 148), and the action of specific endopeptidases (146, 147, 149). Stress conditions profoundly impair these processes. Endoplasmic reticulum stress disrupts the export of properly folded proinsulin, while oxidative stress affects the integrity of granule membranes and interferes with condensation of insulin crystals (16, 86, 150).

Electron microscopy studies of human β-cells from individuals with autoantibodies or early T1D reveal reduced numbers of mature insulin granules, increased numbers of immature vesicles, and abnormalities in granule morphology (16, 110). These defects result in impaired glucose stimulated insulin secretion and increased release of incompletely processed peptides (151, 152). The presence of immature granules also increases the likelihood of aberrant peptides entering antigen processing pathways, contributing to altered immunogenic identity (86, 153, 154).

These secretory abnormalities highlight the interconnectedness of β-cell biology and immunogenicity. Impairments in granule formation may influence the availability of peptide substrates for antigen processing and are therefore plausible contributors to the evolving antigen landscape, although direct evidence establishing necessity or sufficiency in human β-cells remains limited.

## 4 β-cell–derived signals shaping immune activation

### 4.1 Production of cytokines and chemokines by stressed β-cells

β-cells under stress produce several chemokines and cytokines that influence immune cell migration and activation (155, 156) (**Figure 1**). CXCL10 is central among these mediators. β-cells exposed to metabolic or inflammatory stress significantly upregulate CXCL10 (10), which binds CXCR3 on T lymphocytes (156, 157). Increased expression of CXCL10 has been identified in β-cells from individuals with early T1D (156) (**Figure 1**). Spatial mapping studies show that expression of this chemokine corresponds to areas of early insulitis (156).

Other cytokines produced by β-cells include IL-1β and additional inflammatory mediators that reinforce local immune activation (38, 158). These cytokines contribute to oxidative stress, mitochondrial strain, and endoplasmic reticulum dysfunction (114, 130, 159). This β-cell driven inflammatory response is a key feature of the evolving microenvironment and supports the idea that β-cells take an active part in shaping immune activity (86).

### 4.2 Enhanced antigen processing and presentation during β-cell stress

β-cell stress induces profound remodeling of the major histocompatibility complex class one (MHC-I) antigen presentation pathway, fundamentally altering how endocrine cells are surveyed by the adaptive immune system (28, 81). Cytokine signaling, particularly through IFN-γ, robustly increases transcription of MHC-I genes along with proteasomal components and peptide-loading chaperones, resulting in a significant increase in peptide–MHC complexes displayed at the β-cell surface (28, 159). This coordinated upregulation increases β-cell immune visibility and is consistent with enhanced susceptibility to CD8^+^ T-cell recognition under inflammatory conditions.

**Figure 2:**
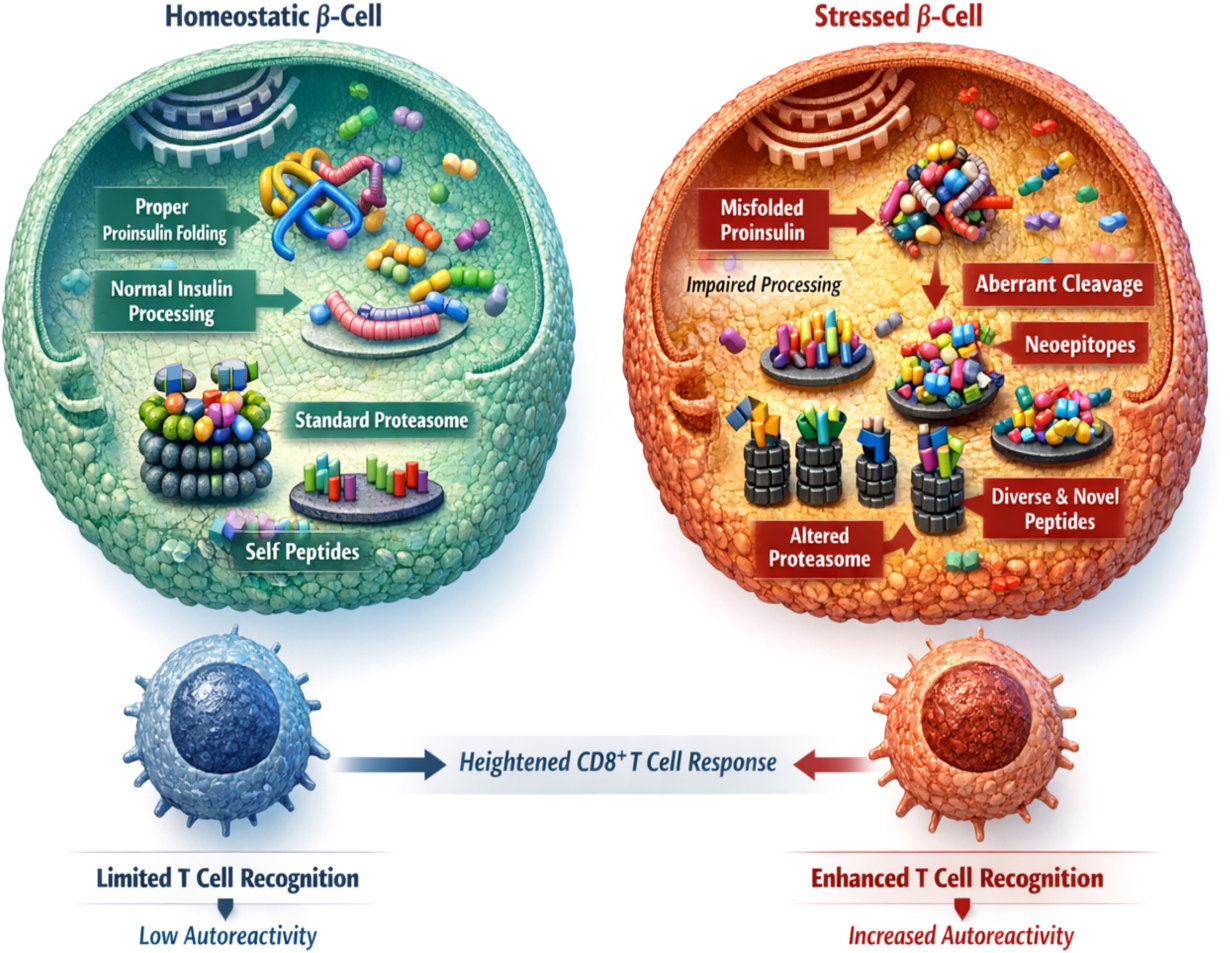
Stress-induced remodeling of the β-cell immunopeptidome. A schematic comparison of homeostatic and stressed β-cells demonstrating stress-dependent changes in antigen generation and presentation. In homeostatic β-cells, efficient proinsulin folding and regulated proteolytic processing produce a limited set of self-peptides presented by MHC class I molecules. Under stress conditions, disruptions in proteostasis alter proinsulin folding and cleavage, modify proteasome and peptide-loading pathways, and promote the generation of non-canonical peptides. These changes result in an expanded and diversified repertoire of MHC-I–bound peptides, increasing the likelihood of recognition by autoreactive T cells. Created in BioRender. Mittal, R. (2026) https://BioRender.com/2a6dcqs.

In addition to increasing antigen abundance, β-cell stress qualitatively reshapes the immunopeptidome. Disruptions in protein folding, trafficking, and degradation broaden the spectrum of intracellular peptides available for presentation, increasing the likelihood that stress-derived and noncanonical peptides are loaded onto MHC-I molecules (28, 101). These changes expand antigenic diversity and enhance the probability of recognition by autoreactive CD8^+^ T lymphocytes (38, 81) (**Figure 2**).

A central contributor to this altered antigenic landscape is proinsulin dysmetabolism, which represents one of the earliest and most consistent biochemical manifestations of β-cell stress (160–162). Impaired folding and processing of proinsulin generates misfolded or partially processed intermediates that are routed toward antigen-processing pathways not typically engaged under physiological conditions (101, 163) (**Figure 2**). These aberrant insulin-derived species serve as a rich source of stress-associated peptides that are preferentially incorporated into the expanding MHC-I peptide repertoire (154, 164–166) Human immunological studies confirm the relevance of this process, demonstrating that both conventional and neo-antigenic insulin-derived peptides are recognized by circulating CD8^+^ T cells in individuals with T1D and in those at elevated risk prior to clinical onset.

The clinical significance of this mechanistic link is highlighted by longitudinal cohort studies showing that elevated proinsulin-to-C-peptide ratios precede overt metabolic dysfunction and strongly associate with progression to T1D (33, 34, 54, 167–169). These observations position proinsulin dysmetabolism as both a biomarker of early β-cell stress and a functional driver of enhanced antigen presentation. Ongoing prevention trials increasingly incorporate proinsulin-based measures as mechanistic endpoints (33, 34, 170, 171).

Together, stress-induced remodeling of antigen-processing pathways and proinsulin dysmetabolism converge to lower the threshold for immune recognition of β-cells. By simultaneously increasing antigen abundance and diversifying antigenic content, stressed β-cells amplify their own visibility to cytotoxic T cells, establishing a direct mechanistic bridge between intrinsic cellular dysfunction and adaptive immune activation. This integrated framework provides a coherent explanation for how early β-cell stress translates into immune engagement and identifies antigen presentation and proteostasis as critical targets for intervention during the preclinical stages of T1D.

## 5 Immunopeptidome diversification in T1D

### 5.1 Conceptual definition of immunopeptidome diversity

In this manuscript, immunopeptidome diversification refers to coordinated quantitative and qualitative remodeling of the peptide repertoire presented by MHC-I molecules on pancreatic β-cells (28, 81, 172). Diversity encompasses an increase in the total number of unique peptide–MHC complexes displayed at the cell surface, alterations in the relative abundance and hierarchy of individual peptide species (173–175). It also refers to the emergence of stress associated or noncanonical peptides generated through aberrant protein folding, altered proteolytic processing, defective granule maturation, or disrupted proteostasis (84, 172, 176–178). Diversification therefore extends beyond simple upregulation of MHC-I surface density and instead reflects a broader transformation in the composition, complexity, and antigenic properties of the β-cell surface.

Under physiological conditions, β-cells present a relatively constrained repertoire derived primarily from normal turnover of intracellular proteins (81, 179). However, when cellular homeostasis is perturbed by endoplasmic reticulum stress, mitochondrial dysfunction, or impaired proinsulin processing, the substrate pool available for antigen processing is altered (172, 180). Misfolded proteins, incompletely processed intermediates, and aberrantly routed fragments become increasingly available for proteasomal degradation and MHC-I loading (181). As a consequence, both the breadth and qualitative features of presented peptides expand beyond the steady state baseline (28, 182, 183). Immunopeptidome diversification thus denotes a stress associated shift in antigenic identity that increases β-cell immune visibility at a molecular level (184–187).

### 5.2 Mechanistic evidence from cytokine-stressed human islets

Direct mechanistic evidence for immunopeptidome diversification derives from immunopeptidomic analyses of human islets exposed to proinflammatory cytokines (28). Interferon signaling induces coordinated transcriptional upregulation of MHC-I genes and key components of the antigen processing machinery, including proteasomal subunits and peptide loading chaperones. This response results in a substantial increase in the number of MHC-I bound peptides detectable at the β cell surface. Importantly, these changes are not limited to quantitative expansion. Cytokine exposure also reshapes the qualitative composition of the peptide repertoire, favoring enhanced presentation of insulin derived sequences and other β-cell specific peptides, including species consistent with altered processing or proteostatic imbalance (28).

Parallel experimental studies demonstrate that endoplasmic reticulum stress and impaired proinsulin folding increase the generation and routing of aberrant peptide fragments into antigen processing pathways. In this context, proteostatic disruption provides a mechanistic link between intrinsic β-cell stress and altered peptide presentation. The combined effect of inflammatory signaling and proteostasis imbalance is therefore a restructured immunopeptidome characterized by both increased abundance and modified peptide identity. These findings suggest that stress may be sufficient to remodel the antigenic landscape of human β cells under controlled conditions and provide a causal basis for the concept of diversification.

### 5.3 Evidence in preclinical autoantibody-positive individuals

Although comprehensive peptide level mapping of the β-cell immunopeptidome in autoantibody positive individuals prior to immune infiltration remains technically constrained by limited tissue availability, several convergent *in vivo* observations support early remodeling of antigen processing pathways (38, 87, 188). Transcriptomic analyses of islets from autoantibody positive donors demonstrate activation of unfolded protein response pathways, interferon signaling signatures, and altered translational programs before overt hyperglycemia (80, 92, 189). These molecular changes are consistent with the intracellular conditions known to promote altered peptide generation and MHC- I loading in experimental systems.

Longitudinal cohort studies further demonstrate that elevations in the proinsulin to C peptide ratio precede clinical diagnosis and correlate with progression risk, indicating persistent proinsulin dysmetabolism during the preclinical phase (33, 34, 190). As impaired proinsulin folding and incomplete processing increase the likelihood of alternative peptide generation, these metabolic abnormalities provide a plausible substrate for diversification of the presented repertoire (177, 184, 191). In addition, circulating CD8 positive T cells recognizing conventional and neoantigenic insulin derived peptides are detectable in individuals at increased risk for T1D prior to clinical onset (185, 192). While these findings do not yet constitute direct immunopeptidomic evidence from early stage human β-cells, they collectively support the presence of stress associated antigen recognition and are consistent with early remodeling of antigen presentation *in vivo*.

### 5.4 Temporal relationship to immune infiltration

Establishing the temporal sequence of immunopeptidome diversification relative to immune cell recruitment is essential for evaluating causality (81, 174). Human tissue analyses indicate that markers of endoplasmic reticulum stress and interferon response can be detected in β-cells from autoantibody positive donors even in the absence of extensive insulitis (80, 87, 193). This observation suggests that elements of antigen processing remodeling may begin before substantial immune infiltration (194–197). At the same time, once autoreactive T cells enter the islet microenvironment, interferon mediated signaling is expected to amplify MHC-I expression and further diversify the peptide repertoire (198–200).

The available evidence therefore supports a dynamic temporal model rather than a strictly linear sequence (80, 87). Early intrinsic β-cell stress may initiate qualitative shifts in peptide generation and presentation, thereby lowering the threshold for T cell engagement. Subsequent immune derived cytokines then intensify and stabilize this remodeled antigenic state, reinforcing immune recognition. Definitive resolution of this sequence will require longitudinal integration of immunopeptidomics, spatial profiling, and single cell analyses across defined stages of human disease in future studies.

## 6 Novelty, testable predictions, and translational implications of the proposed framework

This framework integrates findings with varying levels of mechanistic validation and distinguishes experimentally supported pathways from associations that require further functional confirmation. Rather than viewing β cell stress and immune activation as parallel or sequential phenomena, this model specifies a causal relationship in which stress induced alterations in proteostasis and proinsulin metabolism directly modify both the abundance and diversity of peptides presented by MHC-I molecules. This integration provides a mechanistic bridge between early β-cell dysfunction and the initiation of autoreactive CD8^+^ T cell responses.

A key advance of this framework is that it generates explicit and falsifiable predictions. The model predicts that β cells exhibiting early markers of stress including activation of the unfolded protein response UPR impaired proinsulin processing and elevated proinsulin to C peptide ratios will display a quantitatively expanded and qualitatively altered MHC-I immunopeptidome prior to substantial immune cell infiltration. This prediction can be directly tested using immunopeptidomic and transcriptomic analyses of human islets stratified by β-cell stress burden or disease stage. The framework further predicts that experimental interventions which restore proinsulin folding efficiency or normalize granule biogenesis will reduce the presentation of stress associated insulin derived peptides and attenuate CD8^+^ T cell recognition even under inflammatory conditions. Conversely selective disruption of proinsulin processing is predicted to be sufficient to enhance antigenic diversity and immune visibility in otherwise minimally inflamed β-cells.

This perspective also advances the field by translating cohort level biomarker associations into operational biological criteria that can be evaluated experimentally and clinically. Longitudinal studies demonstrate that elevations in the proinsulin to C peptide ratio subtle impairments in glucose stimulated insulin secretion and early interferon response signatures precede clinical disease onset. Within the proposed framework these readouts are interpreted as indicators of a biologically defined preclinical state characterized by active remodeling of antigen processing pathways. We propose that individuals classified as stage 1 or early stage 2 type 1 diabetes can be further stratified based on β-cell stress biomarkers to identify subgroups with differing degrees of antigenic remodeling and immune engagement. This stratification generates testable hypotheses regarding heterogeneity in disease progression and differential responses to disease modifying therapies.

Importantly the framework delineates specific knowledge gaps that require targeted investigation. These include identification of the antigen processing components most sensitive to distinct forms of β-cell stress determination of whether different stressors generate overlapping or distinct immunopeptidomes and clarification of how β cell heterogeneity influences antigen presentation at the islet level. Addressing these questions will require integrated approaches combining single cell analyses immunopeptidomics and functional T cell assays in human tissue and longitudinal clinical samples.

By defining a causal pathway linking β-cell stress to immune recognition this framework moves beyond a conceptual synthesis and establishes a testable model with clear experimental and translational implications. It positions β-cell intrinsic biology as an active determinant of early autoimmune engagement and provides a structured foundation for biomarker driven risk stratification and intervention during the earliest stages of type 1 diabetes progression.

## 7 Human studies capturing the pre-immune phase of disease

### 7.1 Human islet studies showing early β-cell stress pathways

Human islet studies using organ donor tissue provide direct insights into early β-cell alterations (9, 15). Transcriptomic analyses show that β-cells from individuals with autoantibodies exhibit gene expression patterns consistent with activation of stress and inflammatory pathways (15). Early changes include alterations in the expression of chaperones, oxidative stress genes, antigen presentation components, and inflammatory mediators. Morphological studies reveal disrupted granule architecture and reductions in mature insulin content (154).

### 7.2 Clinical cohort studies provide translational validation

Large longitudinal cohorts such as TEDDY, TrialNet, and various European birth cohorts have enabled detailed characterization of the natural history of preclinical T1D (201, 202). Integration of these metabolic biomarkers with immune markers provides a comprehensive understanding of risk stratification.

Cohort based research confirms that β-cell stress is detectable and clinically meaningful (203). Individuals exhibiting persistent stress biomarkers progress more rapidly than those without such signatures (201). This observation underscores the need to incorporate β-cell centric biomarkers into early detection strategies.

### 7.3 Population screening and implications for public health

The identification of β-cell stress biomarkers supports broader population screening initiatives (204). Early detection allows for initiation of preventive strategies during a window when β-cell mass is still substantial (205). Screening individuals with genetic risk based on human leukocyte antigen profiles and incorporating β-cell centric biomarkers can improve identification of those who would benefit from targeted intervention (34).

Integration of β-cell biomarkers into public health frameworks may allow earlier reclassification of individuals at elevated risk, informing follow up intervals, metabolic testing, and preventive counseling (11) (**Supplementary Table 2**).

## 8 Systems-level perspectives

### 8.1 Systems level interactions between β-cells and immune cells

β-cells reside within a highly integrated islet microenvironment that includes endothelial cells, resident immune cells, neuronal fibers and structural matrix components (206, 207). Early β-cell stress does not remain confined to the endocrine compartment (208). Instead, it initiates a coordinated response across multiple cell types that collectively reshape the immunological landscape of the islet (16, 154). Stressed β-cells alter endothelial behavior by influencing the expression of adhesion molecules and vascular permeability, which can modulate immune cell entry into the tissue (209). Resident macrophages and dendritic cells respond to β-cell derived signals by shifting toward phenotypes that increase inflammatory tone and enhance antigen surveillance.

Metabolic dysfunction within β-cells also produces changes that extend beyond classical cytokine signaling (210, 211). Mitochondrial strain can generate metabolites and redox changes that influence the physiology of neighboring endocrine cells and reshape local cellular communication in parallel (212).

### 8.2 Extracellular vesicles as mediators of β-cell stress propagation beyond the endocrine compartment

While classical microenvironmental interactions within the islet have been largely conceptualized as short-range processes involving soluble cytokines, endothelial modulation, and direct contact with resident myeloid cells, accumulating evidence indicates that stressed β-cells also communicate through extracellular vesicles that enable dissemination of stress signals beyond immediate cellular neighbors (61, 157, 213–215). Small extracellular vesicles, including exosomes, are membrane-enclosed particles released through endosomal pathways that encapsulate defined molecular cargo derived from the parent cell (216–219). Under conditions of metabolic strain or inflammatory exposure, β-cells increase the release of vesicles enriched in chemokines, immune regulatory proteins, stress-associated molecules, and β-cell–specific constituents (215). This vesicular export mechanism provides a protected and spatially unrestricted route by which intracellular perturbations are transmitted to surrounding and potentially distant cellular targets (220–222).

Recent studies demonstrate that proinflammatory β-cell–derived extracellular vesicles contain bioactive mediators such as CXCL10, which can activate CXCR3-dependent pathways in recipient cells and amplify leukocyte recruitment (157, 223, 224). In contrast to freely diffusible cytokines, vesicle-associated chemokines are delivered in concentrated, membrane-protected complexes that may enhance stability and functional potency within the islet interstitium (225, 226). Through uptake by endothelial cells, resident macrophages, and dendritic cells, these vesicles can alter adhesion molecule expression, shift antigen-presenting cell phenotype, and reinforce local inflammatory milieu (61, 227, 228). In this manner, extracellular vesicles extend the influence of β-cell stress from localized paracrine gradients to coordinated, multi-cellular reprogramming across the islet microenvironment (229, 230).

Beyond chemokine delivery, vesicles derived from stressed β-cells may contribute directly to antigenic communication (39, 231, 232). Vesicular cargo can include β-cell proteins and peptide fragments generated under conditions of proteostatic imbalance, thereby providing substrates for cross-presentation by professional antigen-presenting cells (233). Uptake of β-cell–derived vesicles by resident myeloid populations offers a plausible mechanism by which intrinsic endocrine stress is translated into enhanced antigen processing, co-stimulatory molecule expression, and subsequent T cell activation. This pathway supports a model in which antigenic remodeling is not confined to β-cells themselves but is propagated through vesicle-mediated transfer of stress-associated molecular information.

Importantly, extracellular vesicle signaling operates in concert with classical cytokine pathways rather than as a redundant system (61, 157, 234, 235). Soluble mediators establish inflammatory gradients and rapidly modify cellular responsiveness, whereas vesicles deliver complex molecular assemblies capable of inducing sustained transcriptional and functional changes in recipient cells (236, 237). The integration of these signaling modalities allows stressed β-cells to orchestrate both immediate and prolonged alterations in the islet immune landscape (70, 157). Through combined chemokine production, enhanced antigen presentation, and vesicle-mediated cargo transfer, β-cells generate a distributed inflammatory network that may reshape endothelial permeability, promote leukocyte trafficking, and influence antigen-presenting cell activation beyond the immediate endocrine compartment.

Incorporating extracellular vesicle biology into the systems-level framework therefore reinforces the central premise that β-cell intrinsic stress has effects that extend beyond local neighbor-cell interactions. By enabling long-range propagation of inflammatory and antigenic signals, vesicles provide a mechanistic bridge between intracellular proteostatic disruption and broader immune system engagement during the earliest stages of T1D evolution.

### 8.3 Heterogeneity within the β-cell population

Human β-cells display significant diversity in transcriptional identity, metabolic adaptability and sensitivity to stress (238, 239). This inherent heterogeneity allows individual β-cells to respond differently to environmental or inflammatory challenges (240). Some β-cells activate adaptive programs that maintain mitochondrial function and sustain protein folding capacity, whereas others transition toward dysfunctional states that show reduced secretory competence and enhanced immunogenicity (154, 241). These divergent responses generate a mosaic of cellular phenotypes within the same islet.

The dynamic nature of this heterogeneity becomes particularly evident during early disease development. Stress can induce intermediate β-cell states that differ markedly from either healthy or fully dysfunctional phenotypes (80, 141, 242). These transitional cells may possess unique patterns of antigen processing, altered responsiveness to cytokines, or modified communication with neighboring endocrine cells (13, 243). The existence of such states provides a biological explanation for the patchy pattern of insulitis observed in human tissue and the variable decline in β-cell function seen clinically (244, 245). Understanding the molecular determinants that sustain resilient β-cell populations may guide the design of therapeutic strategies that preserve functional endocrine mass during the earliest phases of T1D.

## 9 Therapeutic implications

### 9.1 Redefining the early window of intervention

Evidence that β-cell stress precedes substantial immune infiltration reshapes the conceptual framework of early T1D (106). The preimmune phase can now be viewed as an active pathogenic interval during which β-cell dysfunction contributes directly to the establishment of an autoimmune environment (101, 246). This recognition identifies a critical treatment window in which therapies designed to stabilize proteostasis, reduce metabolic strain or mitigate aberrant antigen presentation may interrupt the early sequence of events that lead to immune activation (247, 248).

Preventive studies are beginning to incorporate biomarkers that reflect these early disturbances, including markers of proinsulin dysmetabolism and subtle defects in glucose stimulated insulin secretion (37, 249–251). Intervening at the early stage has the potential to prevent the amplification of immune responses and preserve β-cell subpopulations that remain functionally intact (252, 253). As mechanistic understanding continues to advance, the early window of intervention is likely to become a central focus of therapeutic development with the goal of modifying disease trajectory before irreversible immune mediated injury occurs.

### 9.2 Therapeutic strategies focusing on β-cell resilience

Therapeutic development in T1D is increasingly shifting toward approaches that reinforce β-cell resilience by stabilizing the molecular systems that govern cellular integrity during early disease evolution (101, 254). β-cells operate at the limits of biosynthetic capacity, and this makes the maintenance of proteostasis a central target for intervention (239). Strategies that strengthen adaptive branches of the unfolded protein response aim to sustain protein folding efficiency and prevent the transition to maladaptive signaling pathways that worsen antigenic remodeling (255, 256). In parallel, the preservation of mitochondrial structure and function has emerged as a critical objective because mitochondria regulate ATP generation, calcium handling and redox balance (257). Each of these processes determines the threshold at which β-cells convert reversible stress into irreversible injury (100, 258). Agents that enhance respiratory chain stability, support the renewal of oxidative phosphorylation components or restore coordinated communication between mitochondria and the endoplasmic reticulum may limit the metabolic instability that promotes early immunogenic transformation (208). Collectively, these metabolic and proteostatic targets reflect a growing recognition that intrinsic β-cell biology is central to shaping the inflammatory landscape of the islet (259, 260).

A second major direction involves strategies that modulate the interface between β-cells and the immune system by influencing the signals that govern immune cell recruitment, activation and recognition (29, 39). Inhibition of stress-induced chemokine programs, particularly those that direct the migration of autoreactive T lymphocytes, may decrease the inflammatory burden placed on vulnerable β-cells (37, 261). Interventions that refine antigen processing within the β-cell aim to reduce the generation of stress derived peptides that are efficiently displayed by MHC molecules and recognized by cytotoxic T cells (37, 262). Recent advances in immunometabolism highlight that β-cell metabolic state directly influences the phenotype of local immune cells, indicating that metabolic therapies can produce secondary immunomodulatory effects within the islet microenvironment (9, 263). This expanding body of work supports a therapeutic framework in which optimal disease modification is achieved through combined stabilization of β-cell intrinsic machinery and targeted modulation of immune pathways (251, 264). Such an integrated approach reflects the mechanistic complexity emphasized in modern immunology and has the potential to alter the natural history of T1D by interrupting the earliest drivers of autoimmune progression.

## 10 A consensus model for β-cell driven autoimmunity

A comprehensive consensus framework becomes evident when mechanistic findings from β-cell biology are integrated with clinical and immunological observations across the natural history of T1D (58, 74). The combined evidence supports a model in which β-cells act as active participants in disease initiation rather than passive targets. Environmental influences, metabolic strain and early inflammatory exposure converge to impose significant stress on β-cells, and this stress disrupts the coordination of proteostasis (204), metabolic regulation and secretory fidelity (39, 249). As β-cells adapt to these challenges, they generate signals that extend far beyond the endocrine compartment (18, 22). These signals include alterations in peptide processing, shifts in organellar communication and changes in the biochemical composition of the extracellular space (18, 22). Collectively, these disturbances reshape the immunogenic profile of the β-cell by modifying the repertoire of presented antigens and altering the cues that regulate immune cell behavior within the islet (265). Immune cells interpret these β-cell generated signals as indications of cellular distress, and their responses initiate the earliest stages of autoimmunity (202). Thus, immune activation is not an isolated event but instead represents a reaction to a progressively dysregulated endocrine environment (18, 111).

The interaction between β-cell dysfunction and immune activation creates a dynamic cycle in which each system amplifies and reinforces the other. Stress-induced alterations in β-cell identity enhance immune recognition, while immune derived cytokines intensify the intracellular disturbances that initially triggered the response (13, 22). Over time this reciprocal escalation produces a self-sustaining state of inflammation that erodes β-cell stability and shifts the tissue ecosystem toward irreversible degeneration (249). This integrated model aligns with the conceptual direction of modern immunology by emphasizing the bidirectional nature of communication between vulnerable tissues and the immune system (81, 101). It also provides a unifying scientific structure that supports the design of next generation therapeutic strategies (28). By clarifying how cellular stress, antigenic remodeling and immune activation intersect, this framework offers critical guidance for mechanistic research, informs selection of biomarkers for clinical monitoring and identifies intervention points that may alter the progression of T1D at stages that precede extensive β-cell loss.

## 11 Future directions and outstanding questions

Although considerable progress has been made in defining the molecular landscape of β-cell stress and its relationship to immune activation, several critical questions remain unresolved and define important priorities for future investigation. A central objective is to distinguish stress-associated alterations that function as pathogenic drivers from those that represent adaptive responses or secondary consequences of inflammation. Observational studies in human tissue have identified extensive remodeling of proteostasis, metabolism, and antigen presentation pathways (16, 80, 266, 267). However, definitive demonstration of causality requires functional perturbation in primary human β-cells coupled with assessment of autoreactive T cell recognition. Establishing necessity and sufficiency for specific stress pathways in modulating immune engagement remains an essential step toward mechanistic clarity.

One particularly important unresolved question concerns the selective vulnerability of β-cells relative to other endocrine cell types within the same inflammatory milieu. In T1D, β-cells are preferentially targeted and destroyed, whereas neighboring α-cells exhibit relative resilience despite exposure to comparable cytokine signals and immune infiltration (268–270). This longstanding observation raises fundamental questions regarding cell type specific differences in antigen presentation, stress adaptation, and immune visibility. Comparative analyses of immunopeptidome composition, MHC-I density, co regulatory molecule expression, and proteostatic capacity between β- cells and α cells may clarify whether intrinsic differences in antigenic repertoire or stress responsiveness account for selective autoimmune targeting. Functional studies examining whether α-cells resist stress induced expansion of antigen presentation pathways or instead engage protective programs that limit immune recognition will be essential to resolve this issue.

Another priority is to define the temporal relationship between β-cell intrinsic stress responses and the initiation of immune infiltration in humans. While cross sectional analyses indicate that stress signatures can be detected prior to extensive insulitis, longitudinal integration of stress biomarkers, spatial transcriptomics, and immune profiling across defined preclinical stages is required to determine whether antigen presentation changes precede autoreactive T cell recruitment or predominantly amplify established inflammation. Advances in high resolution immunopeptidomics and ex vivo functional T cell assays provide an opportunity to directly test these temporal and mechanistic relationships in human tissue.

Finally, greater attention should be directed toward identifying determinants of β-cell resilience. Heterogeneity within the β-cell population suggests that subsets of cells may maintain proteostatic and metabolic stability despite inflammatory exposure. Defining the molecular programs that sustain such resilience, including maintenance of mitochondrial function, preservation of granule integrity, and controlled regulation of antigen processing pathways, may reveal intervention points capable of modifying disease trajectory during the earliest stages of progression.

Addressing these questions will move the field from descriptive characterization toward mechanistic resolution of endocrine cell vulnerability. A systematic focus on causal testing, comparative endocrine biology, and stage specific human investigation will refine understanding of selective autoimmunity and inform the development of therapeutic strategies aimed at preserving β-cell function before irreversible loss occurs.

## 12 Conclusions

Advances across molecular biology, immunology and human tissue analysis have fundamentally reshaped the understanding of T1D by revealing that β-cells are not passive victims of an autonomous immune attack but are instead central determinants of early disease behavior (15). Cumulative evidence indicates that β-cells possess an intrinsic capacity to sense metabolic and inflammatory perturbations and to initiate complex adaptive programs that influence their immunological visibility (81, 241). These adaptive programs involve extensive remodeling of protein quality control pathways, mitochondrial signaling networks and secretory dynamics, and this remodeling generates a spectrum of molecular cues that reshape the surrounding immune environment (21). As these stress induced features accumulate, β-cells produce alterations in antigen generation and chemokine signaling that convey information about cellular fitness to resident and recruited immune cells (15). By recognizing β-cell stress responses and immune activation as interconnected processes, future strategies can target early inflection points that may redirect disease trajectory. This framework supports precision prevention approaches focused on stabilizing β-cell proteostasis, preserving metabolic resilience, and modulating immune pathways responsive to β-cell–derived signals.

The recognition that β-cells actively contribute to the establishment of a proautoimmune environment invites a broader reframing of T1D pathogenesis (10) and opens new conceptual territory for therapeutic intervention. By viewing β-cell stress responses and immune activation as interconnected processes rather than isolated events, it becomes possible to identify early inflection points where targeted intervention may redirect disease trajectory (9, 248). This integrative perspective highlights the potential of therapies that stabilize β-cell proteostasis, maintain mitochondrial resilience, and reduce inappropriate antigen presentation, while also acknowledging the importance of modulating immune pathways that respond to these β-cell derived signals. Such a framework supports the development of biomarkers that capture early cellular instability and offers a scientific foundation for precision prevention efforts. As research continues to converge across disciplines, the emerging model positions β-cell biology as a central pillar in the architecture of T1D and provides a forward-looking direction for future investigation aimed at understanding, predicting and ultimately preventing the progression to clinical disease.

## Data Availability

The original contributions presented in the study are included in the article/**Supplementary Material**. Further inquiries can be directed to the corresponding author.
