# Supplementary material for: Stress-driven remodeling of antigen presentation and chemokine signaling in pancreatic β-cells: implications for type 1 diabetes
Source: Front Immunol. 2026 Apr 23;17:1772399. doi: 10.3389/fimmu.2026.1772399 (PMC13149255; doi:10.3389/fimmu.2026.1772399)
Supplement: Supplementary file 1 [file DataSheet1.pdf]

Supplementary Table 1: β-Cell–Intrinsic Stress Pathways Driving Antigenic Remodeling and Immune Recognition in Type 1 Diabetes

β-Cell Stress Domain	Primary Molecular Perturbation	Downstream Cellular Consequences	Impact on Antigenicity and Immune Recognition	References
Endoplasmic reticulum (ER) proteostasis stress	Chronic proinsulin misfolding and maladaptive unfolded protein response activation	Reduced folding capacity, impaired insulin granule biogenesis, altered Ca^{2+} homeostasis, increased apoptotic susceptibility	Increased generation of non-canonical peptides and enhanced availability for MHC class I presentation	[16, 45, 108, 116]
Mitochondrial metabolic stress and oxidative injury	Impaired oxidative phosphorylation with excess reactive oxygen species production	Decreased ATP generation, impaired glucose–insulin coupling, amplification of ER stress via organelle crosstalk	Oxidative protein modifications broaden the antigenic substrate pool and increase inflammatory sensitivity	[118-122, 126, 129, 131, 136-143, 146]
Vesicle trafficking and insulin granule maturation defects	Golgi dysfunction and impaired proinsulin condensation	Accumulation of immature secretory vesicles, loss of secretory fidelity	Increased entry of aberrantly processed peptides into antigen processing pathways	[16, 86, 110, 147]
Proinsulin dysmetabolism	Inefficient conversion of proinsulin to insulin and C-peptide	Sustained release of incompletely processed proinsulin and early β-cell functional decline	Formation of neo-epitopes preferentially recognized by autoreactive T cells	[33, 34, 54, 160-162]
Stress-induced chemokine and cytokine signaling	β-cell upregulation of CXCL10, IL-1β, and interferon-responsive genes	Establishment of a locally pro-inflammatory islet microenvironment	Directed recruitment and retention of $CXCR3^{+}$ effector T lymphocytes	[22, 156, 157]

β-Cell Stress Domain	Primary Molecular Perturbation	Downstream Cellular Consequences	Impact on Antigenicity and Immune Recognition	References
Antigen processing and presentation machinery upregulation	Interferon-driven induction of MHC class I, proteasome subunits, and peptide-loading complexes	Expansion and diversification of the β-cell immunopeptidome	Increased β-cell visibility to cytotoxic $CD8^{+}$ T cells	[28, 81, 130]
