# Supplementary material for: Stress-driven remodeling of antigen presentation and chemokine signaling in pancreatic β-cells: implications for type 1 diabetes
Source: Front Immunol. 2026 Apr 23;17:1772399. doi: 10.3389/fimmu.2026.1772399 (PMC13149255; doi:10.3389/fimmu.2026.1772399)
Supplement: Supplementary file 2 [file DataSheet2.pdf]

Supplementary Table 2. Integration of β-Cell Stress Biology with Clinical Staging, Biomarkers, and Therapeutic Opportunity in Type 1 Diabetes

Disease Stage	Dominant β-Cell Functional State	β-Cell–Centric Biomarkers	Immunological Consequences	References
Genetically at-risk, pre-seroconversion	Subclinical stress programs with preserved insulin output	Subtle defects in insulin secretory dynamics; early stress-response gene signatures	Minimal immune activation; preserved immune tolerance	[18, 20, 139]
Autoantibody-positive (Stage 1)	Chronically stressed, partially dysfunctional β-cells	Rising proinsulin:C-peptide ratio; impaired first-phase insulin secretion	Enhanced antigen presentation and chemokine-mediated immune recruitment	[16, 20, 28, 33, 79, 80, 156, 159]
Dysglycemia (Stage 2)	Heterogeneous β-cell population with divergent stress tolerance	Elevated proinsulin:C-peptide ratio; declining glucose-stimulated insulin secretion	Escalating bidirectional β-cell–immune inflammatory amplification	[34, 53, 54, 87, 88]
Clinical type 1 diabetes (Stage 3)	Markedly reduced functional β-cell mass with variable residual function	Persistent proinsulin secretion; low but detectable C-peptide	Sustained inflammatory signaling and immune-mediated cytotoxicity	[9, 91, 92, 111, 167]
Post-diagnosis heterogeneity	Inter-individual variability in β-cell survival and stress adaptation	Variable residual β-cell functions across individuals	Explains differential responses to immunomodulatory therapies	[20, 111, 142]
